# Methanol leaf extract of *Azadirachta* *indica* mitigates isoproterenol-induced myocardial infarction through the modulation of oxidative stress, and PPARα and BCL2 signaling in rats

**DOI:** 10.22038/ajp.2024.25277

**Published:** 2025

**Authors:** Amirah Folashade Yusuf, Temitayo Olabisi Ajibade, Oluwaseun Olarenwaju Esan, Racheal Ebunoluwa Asenuga, Moyinoluwa Onoja, Matthew Obot Akpan, Joseph Ayotunde Badejo, Temidayo Olutayo Omobowale, Ademola Adetokunbo Oyagbemi, Adeolu Alex Adedapo, Oluwafemi Omoniyi Oguntibeju, Momoh Audu Yakubu

**Affiliations:** 1 Department of Environmental & Interdisciplinary Sciences, College of Science, Engineering & Technology, COPHS, Texas Southern University, Houston, TX, USA; 2 2Department of Veterinary Physiology and Biochemistry, Faculty of Veterinary Medicine, University of Ibadan, Nigeria; 3 Department of Veterinary Medicine, Faculty of Veterinary Medicine, University of Ibadan, Nigeria; 4 Department of Veterinary Medicine, Faculty of Veterinary Medicine, University of Benin, Benin City, Nigeria; 5 Department of Theriogenology, Faculty of Veterinary Medicine, University of Ibadan, Nigeria; 6 Department of Veterinary Anatomy, Faculty of Veterinary Medicine, University of Ibadan, Nigeria; 7 Department of Pharmacology and Therapeutics, Faculty of Basic Medical Sciences, University of Ibadan, Nigeria; 8 Department of Veterinary Pharmacology and Toxicology, Faculty of Veterinary Medicine, University of Ibadan, Nigeria; 9 Department of Biomedical Sciences, Faculty of Health and Wellness Sciences, Cape Peninsula University of Technology, Bellville, South Africa; 10 Department of Environmental & Interdisciplinary Sciences, College of Science, Engineering & Technology, Vascular Biology Unit, Center for Cardiovascular Diseases, COPHS, Texas Southern University, Houston, TX, USA

**Keywords:** Azadirachta indica, BCL2, Cardiotoxicity, Nephrotoxicity, Isoproterenol, PPARα agonist

## Abstract

**Objective:**

Evaluation of *Azadirachta indica*’s potential on the modulation of blood pressure parameters, antioxidant defense status, as well as immunohistochemical expressions of Peroxisome proliferator-activated receptor α (PPARα) and Bcl-2 (B-cell lymphoma 2) in rats exposed to isoproterenol was the objective of this study.

**Materials and Methods:**

Fifty rats (*Rattus norvegicus*) of the Wistar strain were used, with myocardial infarction induced by intraperitoneal administration of isoproterenol (ISO) for two consecutive days. Cardiac and renal biomarkers of oxidative stress, blood pressure parameters, electrocardiography, and immunohistochemical staining of PPARα and BCL2 were performed.

**Results:**

ISO toxicity heightened blood pressure parameters, aggravated oxidative processes, declined antioxidant defense system, and decreased immunohistochemical expressions of PPARα and BCL2. Interestingly, *A. indica *improved antioxidant status, lowered free radical generation, mitigated serum myeloperoxidase and xanthine oxidase activities, respectively.

**Conclusion:**

Mitigation of oxidative mechanisms and antihypertensive effects of *Azadirachta indica *suggest a positive modulatory role for the medicinal plant in isoproterenol-induced myocardial infarction.

## Introduction

The ever-increasing cardiovascular disease (CVD)-related hospitalization rates and in-hospital mortality continue to post serious challenges to global health (Zhang et al., 2016). CVD includes a range of conditions such as heart attacks (myocardial infarction), strokes and angina. To decrease the chances of CVD occurrence, it is pertinent to modulate in vivo lipid profiles and atherosclerotic pathogenic mechanisms (Taylor et al., 2013). Formation of blood clots may block the artery, a spasm in a coronary artery stops blood flow and can cause a heart attack but this is a rare occurrence (Steven et al., 2013). The arterial wall undergoes structural changes such as hardening and thickening because of accumulated fats, cholesterol, and other compounds (Steven et. al., 2013). Coronary occlusion lasting longer than 30 min causes an irreversible damage to the myocardium thus, resulting in myocardial infarction (Eric et al., 2003).

Isoproterenol (ISO) is a beta-adrenergic agonist that has been reported to induce oxidative stress-mediated myocardial infarction in animal models of CVD (Khalil et al., 2015). ISO-induced cardiotoxicity occurs via free radical generation, energy depletion and hypoxia (Adameova et al., 2009). Moreover, oxidation of ISO metabolites has been implicated in cardiac functional derangement (Bhandari et al., 2008). ISO is an adrenoceptor agonist which can cause cardiac hypertrophy and invariably hypertension after repeated injections in rats (Ren et al., 2019). 

*Azadirachta indica *is a known antioxidant that chelates iron in the body, modulates immune responses and the extract from the leaves reportedly improves the antioxidant defense enzyme system (Shewale et al., 2022). It is proven to be effective against coronary artery disease, hypertension, arrhythmias and several other noncommunicable disease conditions (Sarkar et al., 2021). Presence of various types of biologically active molecules makes *A. indica *effective against various health conditions (Hussain et al., 2023). 

In comparison with some known antioxidants including ascorbate, *A. indica *reportedly confers better potency in its antioxidant mediated effect (Bandyopadhyay et al., 2002; Alzohairy, 2016). An elevated level of antioxidant defense enzymes has been reported in mice post treatment with *A. indica *(Koul et al., 2012 and 2014). In myocardial degeneration, elongation of the QT segment was considerably prevented by the administration of *A. indica *and histopathology of animals co-treated with *A. indica* improved; an indication of the cardioprotective effect of *A. indica* (Ashwani et al., 2014). The evaluation of a probable positive modulatory role for *A. indica *in ISO-induced cardiotoxicity was the aim of this study. 

## Materials and Methods

### Processing of A. indica leaves

The leaves of *A. indica* which were collected fresh underwent cold extraction method. The processing of the leaves of *A. indica* involved rinsing under running water prior to air dying and maceration. Thereafter, one kilogram of the leaves was soaked in eight litres of methanol and allowed to stand for 72 hr with intermittent shaking before filtration and subsequent evaporation to dryness as previously described by Ajibade et al. (2022). 

### Study design

Five groups of rats comprising of 10 rats each were used for this study. The animal groupings included the control, the ISO at 85 mg/kg (Adeoye et al., 2019), 100 mg/kg *A. indica*, 200 mg/kg *A. indica* and 300 mg/kg Clofibrate. Corn oil, *A. indica* and 300 mg/kg Clofibrate , from Sigma (St. Louis, Missouri), were administered orally for 14 consecutive days, whereas ISO was administered for two consecutive days via the intraperitoneal route.

### Ethical approval

The ethical approval for this study was obtained from the Animal Care and Use Research Ethics Committee of the University of Ibadan, Nigeria with approval number UI-ACUREC/003-0718/9.

### Blood pressure determination

This was carried out in non-anaesthetized trained rats in accordance with the previous methods of Omóbòwálé et al. (2018). The apparatus used was a 6/7 lead computer ECG machine, EDAN 1010. The machine was set at 50 mm/s paper speed and 10 mm/mv voltage calibrations. 

### Blood collection and serum preparation

Blood samples were collected into dry clean plain via the media canthus of the eye by careful puncturing of the plexus behind the eye. The blood was allowed to clot in slanting plain sample bottles for 30 min to obtain the sera. 

### Tissue preparation for biochemical assays

Following the experimental period, the hearts and kidney tissues were harvested from humanely sacrificed rats. The rats were sacrificed by cervical dislocation following anesthesia with xylazine and ketamine (0.1 mL/100g; v/v). The tissues were rinsed in ice-cold 1.15% Potassium Chloride (KCl) and weighed. The weights of the organs were recorded for analysis. Thereafter, extraction of the post mitochondria fraction (PMF) was done by homogenizing the tissues in phosphate buffer, and cold centrifuging the homogenate.

### Biochemical assay

#### Oxidative stress biomarkers

Malondialdehyde and hydrogen peroxide were analyzed in the heart and kidney tissues using standard methods previously reported by Varshney et al. (1990), and Wolff 1994), respectively. Also, myeloperoxidase (MPO) was analyzed in the sera of rats (Xia et al., 1997). 

#### Antioxidant status

The indices of antioxidant defense status analyzed in this study included the antioxidant enzymes such as superoxide dismutase (SOD), glutathione S-transferase (GST), and glutathione peroxidase (GPx), and the non-enzymatic antioxidant, reduced glutathione (GSH). SOD was analyzed as described by Kirby and Fridovich (1982), GST according to the method of Keen et al. (1976), and GPx was assayed based on the method of Wang et al. (1994). The level of GSH in the cardiac and renal tissues was assayed as described previously (Beutler et al., 1963).

### Immunohistochemical analysis of Peroxisome proliferator-activated receptor – alpha (PPAR)

The immunohistochemical analysis of the heart and kidney tissues was carried out as previously described (Oyagbemi et al., 2020). Briefly, the cardiac tissue was sectioned and cleaned properly. The sections were cleaned with tissue paper, goat serum was added, and the slides were incubated in a humidifying chamber for 15 min. After the incubation, the slides were shaken to remove excess goat serum, and probed with anti-PPARα and anti-BCL_2_ polyclonal antibodies for heart and kidney tissues, respectively, (ABclonal Biotechnology co., Ltd). After 12 hr, the slides were placed in wash buffer tank for 5 min, removed and covered with goat anti-rabbit serum. Subsequently, the slides were incubated with Horseradish Peroxidase, rinsed with phosphate buffer saline and substrate diaminobenzidine (DAB) was added. Termination of reaction was achieved with the addition of distilled water and slides were counterstained with haematoxylin for 3 sec. 

### Statistical analysis

Were applicable, the data from this study were analyzed with the Student’s T test and analysis of variance using a statistical package Graphpad Prism version 5.0, which was used for the plotting all graphs. The value of probability p<0.05 was considered statistically significant.

## Results

### Organ weights

**Figure 1:**
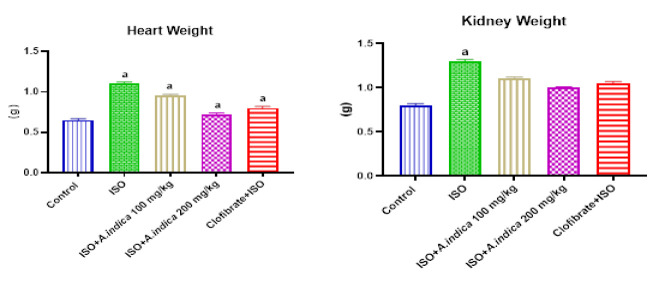
Organ weight. Values are presented as mean±standard deviation (n=10). a=Significant (p<0.05) increase when compared with the control group, b=p<0.05 when compared with the ISO group.

Figure 1 shows a significant increase in weight of the heart and kidney tissues for ISO group in contrast to the control. Moreover, the weight of these organs was higher in *A. indica* and Clofibrate groups compared with the ISO-only group. 

### Hydrogen peroxide and malondialdehyde levels

**Figure 2:**
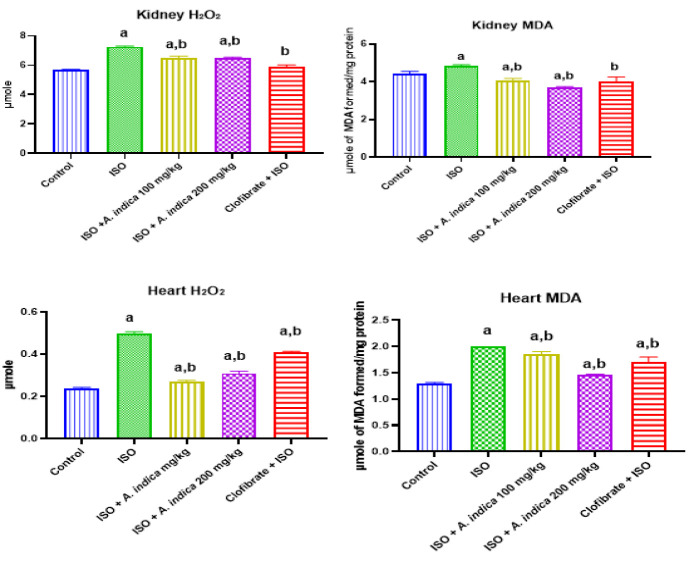
Hydrogen peroxide (H_2_O_2_) generation and malondialdehyde content in the heart and kidneys. Values are presented as mean±standard deviation (n=10). a=significant (p<0.05) increase when compared with the control group, b=significant (p<0.05) decrease when compared with the ISO group.

Figure 2 shows that ISO increased H_2_O_2_ level of cardiorenal tissues relative to the control group. However, *A. indica *decreased H_2_O_2_ levels of heart and renal tissues relative to the ISO-treated rats. Furthermore, MDA content of the heart and kidney in ISO-treated rats increased significantly (p<0.05) compared with the control. However, MDA level for *A. indica *relative to ISO only treated group. 

### Enzymic and nonenzymic antioxidants

**Figure 3:**
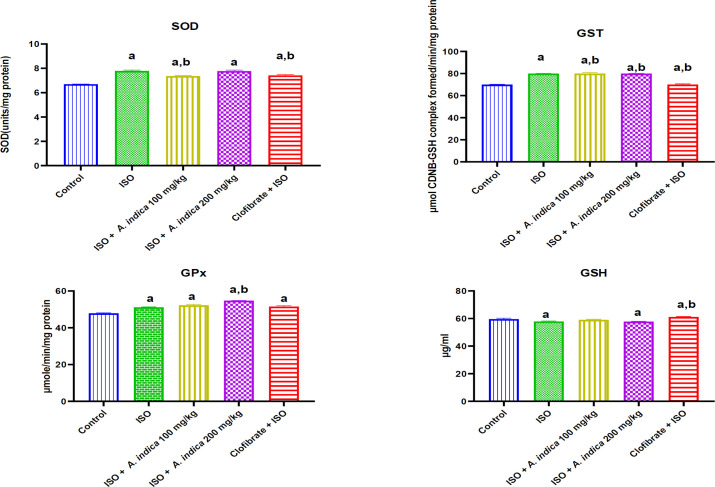
The effect of *A. indica* on antioxidant activity in the cardiac tissue of rats exposed to ISO. Values are presented as mean±standard deviation (n=10). a = p<0.05 when compared with the control group, b = p<0.05 when compared with the ISO group. GSH (reduced glutathione GPx: Glutathione peroxidase: GST: Glutathione S-transferase).

**Figure 4:**
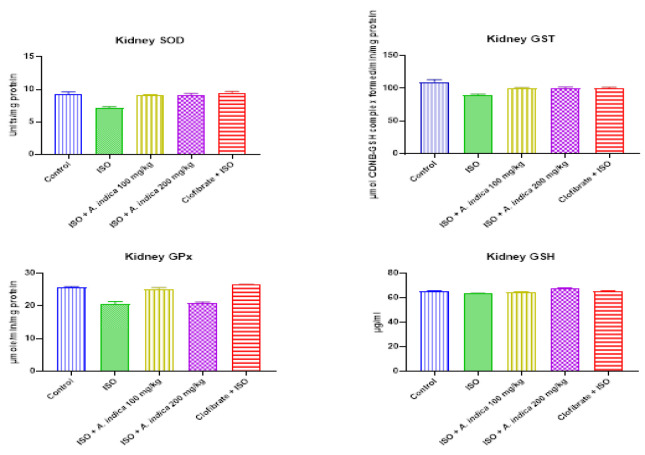
The effect of *A. indica* on antioxidant activity in the renal tissue of rats exposed to ISO. Values are presented as mean±standard deviation (n=10). a=p<0.05 when compared with the control group, b=p<0.05 when compared with the ISO group. GSH (reduced glutathione GPx: Glutathione peroxidase: GST: Glutathione S-transferase).

The results in Figure 3 show that there was a decrease with a significant (p<0.05) difference in the cardiac and renal GSH level in the ISO treatment group when compared with control group. Treatment groups *A. indica* at 100 mg/kg and 200 mg/kg show there was an increase in cardiac and renal GSH level when compared with ISO group. However, the result showed that there was a significant increase in the cardiac SOD, GPx and GST activity of the ISO group when compared with the control. Figure 4 shows that there was a significant decrease in the renal SOD activity of the ISO group when compared with the control. There was also an increase with a significant (p<0.05) difference in the renal SOD activity of the treatment groups of *A. indica* at 100 mg/kg and 200 mg/kg compared with the ISO group. Renal glutathione transferase activity was decreased with a significant difference (p<0.05) in the ISO treatment group when compared with the control group. The treatment groups of *A. indica* at 100 mg/kg and 200 mg/kg showed an increase in renal GST activity relative to ISO group. Furthermore, there was a decrease with a significant difference (p<0.05) in renal GPx activity in the ISO group when compared with the control group. However, pre-treatment with *A. indica* 100 mg/kg caused a significant increase (p<0.05) in GPx levels when compared with the ISO group.

### Serum marker of inflammation

**Figure 5:**
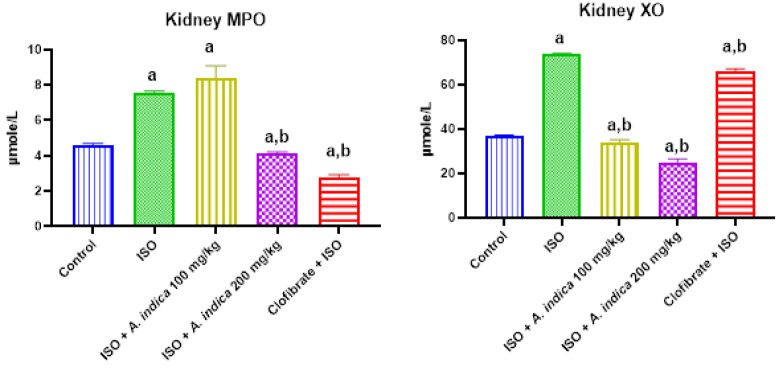
The effect of *A. indica* on serum markers of oxidative stress of rats exposed to ISO. Values are presented as mean±standard deviation (n=10). a=p<0.05 when compared with the control group, b=p<0.05 when compared with the ISO group. MPO = Myeloperoxidase; XO = Xanthine oxidase.

Figure 5 shows that there was a significant increase in the MPO activity of the ISO group when compared with the control. There was also a decrease with a significant difference (p<0.05) in the MPO activity of the *A. indica* at 100 mg/kg and 200 mg/kg compared with the group B. Results from this work also showed a marked increase with a significant difference (p<0.05) in serum xanthine oxidase (XO) activity in the ISO group when compared with the control group. Furthermore, pre-treatment with 100 and 200 mg of *A. indica* caused a significant decrease in the serum XO levels when compared with the ISO only treated group. 

### Blood pressure parameters

**Figure 6:**
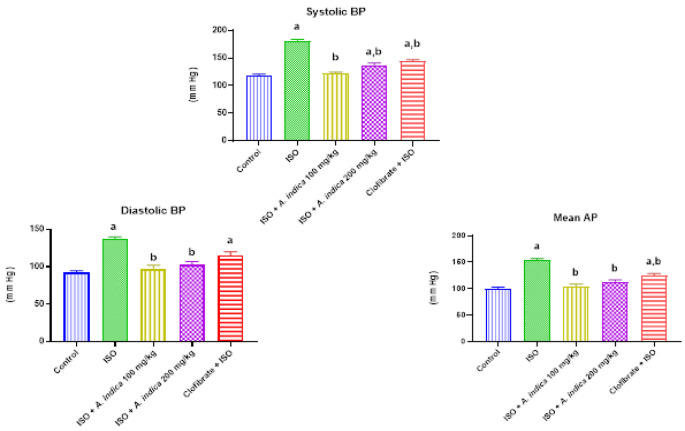
The effect of *A. indica* on blood pressure parameters of rats exposed to ISO. Values are presented as mean±standard deviation (n=10). a=p<0.05 when compared with the control group, b=p<0.05 when compared with the ISO group. BP = Blood Pressure; AP = Arterial Pressure.

Figure 6 shows that the blood pressure measurement in the ISO group significantly increased compared to the control. There was also a decrease with a significant difference (p<0.05) in blood pressure measurement of the treatment groups of *A. indica* at 100 mg/kg and 200 mg/kg compared with the ISO group. 

### Immunohistochemistry

**Figure 7.**
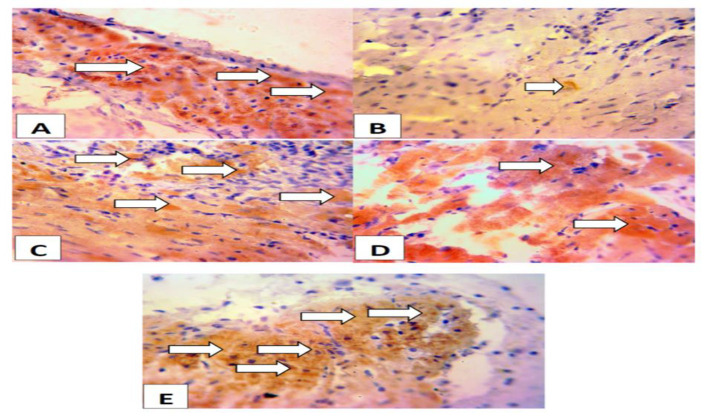
Immunohistochemistry of Peroxisome proliferator-activated receptor (PPAR)-alpha in the cardiac tissues. Group A (control), Group B (ISO; 100 mg/kg), Group C (ISO + AI 100 mg/kg), Group D (ISO + AI 200 mg/kg), Group E (ISO + Clorfibrate 300 mg/kg). Rats administered with ISO alone showed lower expressions of PPAR. Whereas higher expressions of PPAR were observed in rats pre-treated with A. indica AI or Clofibrate compared to the control. The slides were counterstained with high-definition Hematoxylin and viewed at X 40 magnification.

Figure 7 shows that rats pretreated with *A. indica *or clofibrate had higher expressions of PPAR compared to the ISO- only group. Lower expression of PPAR was observed in the ISO-only group. Rats administered with ISO alone showed lower expressions of PPAR. Whereas higher expressions of PPAR were observed in rats pre-treated with *A. indica* or Clofibrate compred to the control..

**Figure 8:**
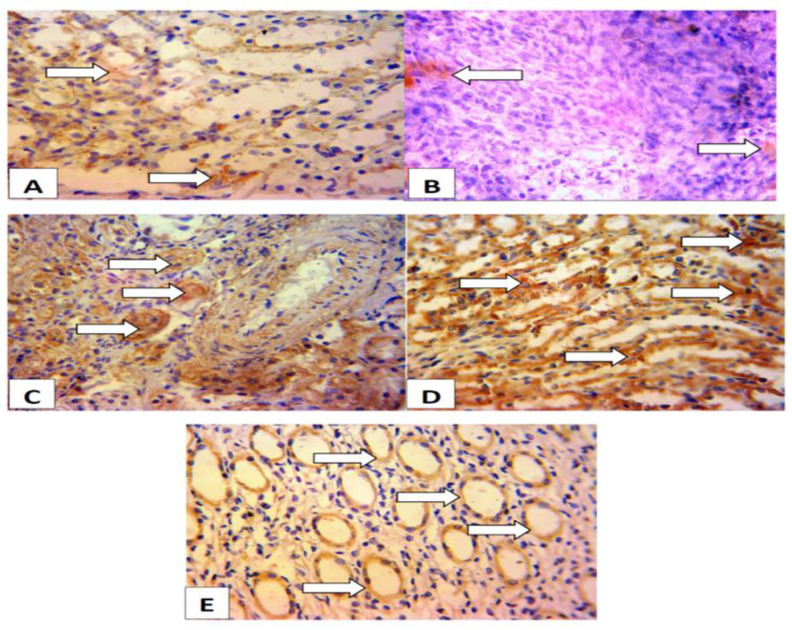
The expressions of BCL2 in the renal tissues. Group A (control) shows higher expressions of BCL2 (anti-apopototic protein) in the renal tissues. Group B (Isoproterenol; 100 mg/kg) shows lower expressions of BCL2. Group C (Isoproterenol + *A. indica* at 100 mg/kg) shows higher expressions of BCL2 than Isoproterenol group. Group D (Isoproterenol + *A. indica* at 200 mg/kg) shows higher expressions of BCL2 compared to Group B. Group E (Isoproterenol + Clorfibrate at 300 mg/kg) shows expressions of BCL2 similar to that of control group. The slides were counterstained with high-definition Hematoxylin and viewed a magnification of x 400.

Figure 8 shows that rats pretreated with *A. indica* or Clofibrate had higher exprssions of BCL2 compared to the ISO-only group. Lower expression of BCL2 was observed in the ISO-only group. 

## Discussion

The phytochemical profile of *A. indica* (Neem) reveals diverse group of chemical compounds with different pharmacological activities (Patil et al., 2022). *A. indica *is believed to possess antioxidant activity (Nahak and Sahu, 2010). Medicinal plants are well recognized sources of antioxidants with diverse array of potentially beneficial medico-pharmacological uses (Martinelli et al., 2021). An elevated level of antioxidant defense enzyme has been reported in mice post treatment with *A. indica *(Koul et al., 2012). Likewise, *A. indica *has been reported to be renoprotective (Gupta et al., 2016), with the extracts from the leaves reported to decrease indicators of renal functional damage such as creatinine and (Kpela et al., 2013).

ISO-induced cardiotoxicity is mechanistically mediated by free radical generation, energy depletion and hypoxia (Adameova et al., 2009). ISO-induced myocardial damage entails membrane permeability alterations resulting in the loss of function and integrity of myocardial membranes (Sudhees et al., 2013). Studies have reported that high doses of ISO for 2 consecutive days at 150 mg/kg resulted in extensive myocardial injury with severe vacuolization in heart muscle and inflammation with diffused inflammatory cell infiltration (Sahu et al., 2016). 

Furthermore, pre-treatment with *A. indica *was able to alleviate the effect of free radicals generated by ISO. This work has demonstrated that *A. indica* has a chemo preventive effect on the cardiotoxicity and nephrotoxicity induced by ISO via the attenuation of oxidative stress. The cardioprotective role of *A. indica *extract has been demonstrated by increases in the activities of antioxidant enzymes, such as GST and GPx (Koul et al., 2014). Lipid peroxidation has been implicated in many disease progression with lipid hydroperoxides as one of its major products (Morita et al., 2016). These molecules are recognized as cytotoxic because they can form covalent adducts with other biomolecules (Larsson et al., 2016). In the present study, treatment with ISO resulted in a significant rise in oxidative stress markers together with decreased heart rate, increased lipid peroxidation and blood pressure, and changes in endogenous antioxidants SOD, GPx, GST and GSH. These changes, however, were ameliorated when the rats were pre-treated with *A. indica *extract. Depletion of intracellular non-enzymic antioxidant defense system (GSH) by ISO administration also contributed to the induction of oxidative stress and the triggering of lipid peroxidation products in the heart and kidney. However, *A. indica* pre*-*treatment inhibited depletion of GSH by ISO in both the heart and kidney. Therefore, it could be inferred that *A. indica *might be involved in the improvement of the GSH recycling system during oxidative damage. 

The present findings demonstrate that methanol extract of the leaves of *A. indica* significantly protected the heart and kidneys, exerting cardioprotective and nephroprotective properties. Oxidative stress is suppressed or delayed by antioxidants which inhibits the oxidative process and *A. indica *is a source of natural antioxidant which can be used for the prevention or treatment of diseases because of oxidative stress (Pangjit et al., 2014). 

Our study revealed that ISO treatment significantly inhibited SOD, GPx, and GST in renal tissues but pre-treatment with the leaf extract of *A. indica *provided some level of protection in the pre-treated groups. The reductions noted in the antioxidant enzyme activity in the kidney were normalized with *A. indica *relative to the ISO-treated rats. Also, SOD, GPx, and GST activity increased significantly in cardiac tissue of ISO-treated rats. The adaptive response could be a result of upregulation of the transcription factor, nuclear factor E2-related factor 2 (Nrf2). Nrf2 is a critical transcription factor that modulates the induction of phase 2 detoxifying and antioxidant genes (Yan et al., 2010). Furthermore, studies have shown that endogenous antioxidants induction through activation of Nrf2 has been revealed to prevent oxidative stress and confers cardio protection (Dreger et al., 2009). 

SOD can be found in most cells and is the first line of defense against ROS and thus is one of the most important enzymes among the antioxidant defense system (Ruth et al., 2002). SOD catalyzes the detoxification of superoxide anion into H_2_O_2_ and water (Miller, 2012). However, GSH and antioxidant enzymes like CAT, GPx, and GST, normally ensure the final decomposition of H_2_O_2 _to water and oxygen (Sharma et al., 2010). 

The increase in serum XO activity is invariably indicative of hyper uricemia. Hyper uricemia has been linked to hypertension and renal failure (Chengfu et al., 2015). The ability of *A. indica* to significantly reduce serum XO is an indication of nephroprotective effect of *A. indica*. Results from this study showed elevated values of blood pressure parameters including the systolic, diastolic and mean arterial pressure in ISO-intoxicated rats, which is evidence of the hypertensive effects of ISO toxicity. However, pre-treatment with *A. indica *ameliorated the hypertensive effect in the rats as previously reported (Yarmohammadi et al., 2021). 

Bcl-2 (B-cell lymphoma/leukemia-2) are proteins which are central regulators of apoptosis (Khan et al., 2018). In nephrotoxicity, acute ischemic injury in rats is characterized by two peaks of apoptosis. The first small peak (at three days) is followed by a rapid decrease in number of apoptotic cells. For three days the Bcl2 expression is usually elevated then later gets back to normal levels (Sharpe et al., 2004). From the results, the control shows elevated expressions of BCL2 in the renal tissues. However, ISO group had reduced expression of BCL2. The increase in the expression of BCL2 in *A. indica* pre-treated rat is suggestive of anti-apoptotic effect of *A. indica*.

Omega-3 fatty acid is a natural ligand of PPARα and when it undergoes oxidation is converted into a stronger PPARα agonist (Volker et al., 2000). It can be speculated from this work that *A. Indica *can serve as a potential PPARα ligand. Nimbolide a substance obtained from *A. indica* has been shown to exhibit anti-tumor properties and has been identified as a potential PPARα ligand (Nagini et al., 2021).

Conclusively, this study has been able to demonstrate that administration of ISO increased lipid peroxidation, generated free radicals, inhibited enzymatic and non-enzymatic antioxidant machinery and inflammatory response. *A. indica *attenuated markers of oxidative stress, improved antioxidant defence system, reduced blood pressure and improved heart rate. Similarly, *A. indica *pre-treatment exhibited cardioprotective and anti-apoptotic property through increase in the expressions of PPARα and BCL2 respectively. Therefore, the leaves of *A. indica* could be employed as a potential phytochemical that could mitigate cardiotoxic disease, and nephrotoxicity and the cardiotoxicity associated with chemotherapeutic agent.
